# Parallels and Mutual Lessons in Tuberculosis and COVID-19 Transmission, Prevention, and Control

**DOI:** 10.3201/eid2703.203456

**Published:** 2021-03

**Authors:** Philip C. Hopewell, Lee B. Reichman, Kenneth G. Castro

**Affiliations:** Zuckerberg San Francisco General Hospital, University of California, San Francisco, California, USA (P.C. Hopewell);; Rutgers New Jersey Medical School, Rutgers University, Newark, New Jersey, USA (L.B. Reichman);; Emory University Rollins School of Public Health, Atlanta, Georgia, USA (K.G. Castro);; Emory University School of Medicine, Atlanta (K.G. Castro)

**Keywords:** COVID-19, coronavirus disease, SARS-CoV-2, severe acute respiratory syndrome coronavirus 2, viruses, respiratory infections, zoonoses, tuberculosis and other mycobacteria

## Abstract

The coronavirus disease (COVID-19) pandemic has had unprecedented negative effects on global health and economies, drawing attention and resources from many other public health services. To minimize negative effects, the parallels, lessons, and resources from existing public health programs need to be identified and used. Often underappreciated synergies relating to COVID-19 are with tuberculosis (TB). COVID-19 and TB share commonalities in transmission and public health response: case finding, contact identification, and evaluation. Data supporting interventions for either disease are, understandably, vastly different, given the diseases’ different histories. However, many of the evolving issues affecting these diseases are increasingly similar. As previously done for TB, all aspects of congregate investigations and preventive and therapeutic measures for COVID-19 must be prospectively studied for optimal evidence-based interventions. New attention garnered by the pandemic can ensure that knowledge and investment can benefit both COVID-19 response and traditional public health programs such as TB programs.

In addition to having devastating effects on the economies of the world, the pandemic of coronavirus disease (COVID-19) itself and the responses entailed in containment and mitigation efforts could have disastrous consequences for existing public health programs, with the impacts being most pronounced in high-burden, low-income settings (*1*,*2*). Modeling of the impact of the COVID-19 pandemic conducted by Imperial College London (London, UK) suggests that in high-burden settings, disease-related deaths over 5 years might be increased by up to 10% for HIV, 20% for TB, and 36% for malaria (*1*).

To minimize the adverse consequences of COVID-19 on overall public health services, synergies between COVID-19 response and traditional public health programs should be sought and the lessons and resources developed in any of the programs should be used for the benefit of the others. In this regard, approaches to TB control might hold lessons for the public health response to COVID-19 and vice-versa.

## Synergies and Commonalities for COVID-19 and TB

Several commonalities exist between COVID-19 and TB, most notably transmission of their etiologic agents, severe acute respiratory syndrome coronavirus 2 (SARS-CoV-2) and *Mycobacterium tuberculosis*. Both pathogens are transmitted through secretions from the respiratory tract (*3*–*5*). Moreover, protecting healthcare workers and other susceptible patients, and contact identification and evaluation are key components of the public health response to both infections. An understanding of the routes of and factors influencing transmission is necessary to develop effective and efficient measures to control the diseases. For TB, many years of clinical and experimental studies have provided a wealth of information on which to base contact identification, prioritization, and evaluation (*4*). Investigations of TB outbreaks have been especially informative (*6*). Not surprisingly, this level of understanding of SARS-CoV-2 transmission does not exist, and the relative contributions to transmission of large respiratory droplets, fomites, and aerosols remain controversial (*7*). Notably, transmission of both pathogens has been associated with superspreader events (*8*–*10*).

The clinical manifestations of COVID-19 were initially described as mainly involving the respiratory tract, with cough as a predominant symptom along with fever, but knowledge of its full natural history, with both immediate and potential long-term consequences, is still increasing rapidly (*3*,*11*). Both the degree of infectiousness and the severity of SARS-CoV-2 infection dictate rapid and effective implementation of healthcare facility infection prevention and control to minimize transmission. These measures include administrative, engineering, and personal measures (i.e., personal protective equipment) and community-based public health activities, even without strong empirical evidence on which to base these interventions.

## Seeking COVID-19 Mitigation and Control Strategies

Unquestionably, the package of community-based mitigation measures put into place for the current pandemic has had a major effect in reducing cases and deaths, as shown by Hsiang et al. (*12*). However, uncertainties remain concerning the most effective individual or combinations of measures. These uncertainties preclude the ability to readily identify more targeted and efficient control strategies. Thus, an urgent need exists for a more detailed understanding of SARS-CoV-2 transmission routes and patterns.

Of particular importance is the implementation of monitoring and rapid case identification as current mitigation measures are relaxed. General agreement exists that rapid case identification through PCR-based testing quickly followed by contact identification and evaluation (generally called contact tracing in the context of COVID-19) is the key strategy in reducing transmission in settings where the epidemic curve is flattened or declining (*13*,*14*). Earlier in the pandemic, after the spring 2020 surge subsided, this approach, which closely resembles strategies used for TB, was being scaled up and implemented rapidly. The core actions involve identifying persons with the disease (index case-patients) and identifying and evaluating persons exposed to the index case-patient (contacts) to find additional cases and offer contacts preventive interventions. However, rapid increases in cases in late fall and winter 2020 made contact tracing impractical, simply because of volume. Now, as the pandemic wanes, a trend that we hope will continue, contract tracing is again becoming feasible.

## Value of Contact Identification and Evaluation

Contact identification and evaluation have been key components of TB-control measures in most low TB–incidence countries for at least the past 75 years, and a strong scientific basis exists for most, but not all, elements of this activity (*15*,*16*). Although the same information and approaches apply in generally resource-poor, high TB–incidence countries and although international guidelines exist, implementation of routine contact investigations has been very limited (*17*,*18*). In the setting of TB, effective contact investigations have addressed stigma, community engagement, training of interviewers, and use of specific operational guidelines (*17*,*19*). These same elements will likely prove crucial to the effectiveness of COVID-19 contact tracing.

At least 3 important differences exist between factors that should be considered when engaging in contact identification and evaluation for COVID-19 compared with TB. First, because of the short interval between exposure and disease onset, estimated to be a median of 4.1 days for COVID-19, the timeframe for contact identification and evaluation is much shorter than for TB (*20*). In addition, infection with *M. tuberculosis* in immunocompetent hosts most commonly results in latent infection, which can last decades and in most cases never progresses to active TB disease. Second, persons with COVID-19 are most infectious in the immediate presymptomatic and early symptomatic phases, when the viral titers are at their peak, again indicating the need for speed in the contact process for maximal effectiveness (*20*). Third, SARS-CoV-2 clearly is transmitted from person to person predominantly through respiratory secretions that may be inhaled, settling on the mucosal lining of large airways, or be self-inoculated onto nasal mucosa or into the eyes (*7*,*11*,*21*). Unlike TB, the droplets with the SARS-CoV-2 viral cargo might also contaminate and persist on surfaces, although the role played by surface or fomite transmission is not well-quantified (*22*). However, increasing controversies and concerns exist as to the relative contribution of aerosols to overall transmission (*7*,*11*).

## The Role of Droplet Nuclei and Acquisition of Infection

*M. tuberculosis* is transmitted nearly exclusively by aerosolized droplet nuclei, particles <5 µm in aerodynamic diameter (*23*). Large droplets per se are not effective vehicles for transmission of *M. tuberculosis*; however, as the water content of large droplets evaporates, droplet nuclei are formed. The closeness and duration of exposure to a person with infectious TB, as well as the ventilation of the space in which the exposure occurs, influence the likelihood of transmission. Nevertheless, TB outbreaks have been documented with more casual exposures in churches, schools, nursing homes, prisons and jails, and long airplane flights, as well as in other congregate settings, many of which have also been locations of documented SARS-CoV-2 transmission (*6*,*23*–*27*).

Direct and indirect evidence that SARS-CoV-2 may also be transmitted by aerosols with droplet nuclei (i.e., fine particles that remain suspended in air) carrying infectious particles (*5*,*7*,*28*) is increasing. A description of an outbreak of COVID-19, associated with a restaurant in Guangzhou, China, strongly suggested transmission through an airborne route (*29*), as did case distribution and additional studies of air circulation, also in this restaurant in Guangzhou (Y. Li, unpub. data, https://doi.org/10.1101/2020.04.16.20067728).

For both TB and COVID-19, cough is a predominant symptom, and airborne droplets are produced by any forced expiratory maneuver, especially coughing; at least for TB, the severity of cough is an indicator of transmission risk. For TB, several additional indicators assist in quantifying the risk for transmission from the index case and, thus, in assigning priority to a contact investigation. These indicators include the bacillary burden, as indicated by the radiographic extent of the disease in the lungs and the presence or absence of cavitary lesions and qualitative sputum smear positivity (*16*,*30*). No such assessment is routinely used for COVID-19, although quantification of viral load in nasal or pharyngeal swab specimens and an assessment of the severity and duration of respiratory symptoms could provide such information (*31*,*32*). Reduction in viral inoculum by widespread wearing of masks has been postulated to result in less severe manifestations of SARS-CoV-2 infection (*33*).

For TB, because of the increasing risk for acquisition of infection with the closeness and duration of exposure to persons with this disease, contact evaluation can be structured, beginning in the home, workplace, or school, and places of leisure and working outward in a manner that conceptually resembles concentric circles. The number and percentage of close contacts with evidence of disease, or recent infection, inform the need to expand the investigation to contacts in outer ring circles. This iterative approach optimizes the use of resources for investigations and testing (*16*,*30*). For SARS-CoV-2, data strongly suggest that the virus is highly transmissible even with casual contact, so the duration of exposure might not be relevant (*14*,*20*,*32*).

All of the foregoing indicates that in conducting contact identification and evaluation for persons exposed to persons with COVID-19, a wide net must be cast. Moreover, given the incubation period and pace of the disease, the process must be accomplished much more quickly than is necessary for TB. Unfortunately, much of the knowledge base that is used to guide TB contact identification and evaluation does not yet exist for COVID-19. To generate the necessary information, investigators studying the epidemiology of COVID-19 and, in particular, those charged with investigating outbreaks and conducting contact tracing, should be certain that the data being collected will enable analyses directed toward identifying factors that influence viral transmission. A recent report of nationwide contact tracing for COVID-19 in South Korea indicated both the need to investigate »10 contacts per index case and that 11.8% of household contacts had COVID-19, >6 times the 1.9% prevalence of COVID-19 in nonhousehold contacts (*34*).

## Using the Investigation of TB on the USS Byrd as a Template

Essentially all infection control and public health measures for TB are based on the understanding, backed by strong empirical and experimental evidence, that *M. tuberculosis* is transmitted nearly exclusively by aerosols (*23*,*35*). Some of the strongest evidence of *M. tuberculosis* transmission through aerosols has been derived from several TB outbreak investigations. Perhaps the most notable and informative outbreak investigation was conducted in response to a single crew member who was found the have cavitary pulmonary TB during the course of a long sea tour by the US Navy vessel the USS Richard Byrd in 1965 (*36*). A thorough assessment of the patterns of air circulation and their relationship to new cases and infections was conducted aboard the ship. The investigation found that all new cases and infections occurred in crew members who had either direct personal contact with the index case-patient or were exposed through recirculated air in a closed ventilation system. The investigators were able to establish what might be viewed as a dose-response curve based on the exposure to different amounts of recirculated air and the proportion exposed crew members who were infected (*36*). Of particular note, several of the newly infected sailors (indicated by a new positive tuberculin skin test) who were asymptomatic and had negative chest radiographs were found to have *M. tuberculosis* in their sputum, raising the possibility of transmission from persons without the usual symptoms of TB, as is the case with COVID-19 (*20*,*32*). This finding is consistent with findings from national TB prevalence surveys of a substantial proportion of study subjects who were found to have *M. tuberculosis* in their sputum but had no symptoms (e.g., cough >2 weeks) (*37*).

Outbreaks of COVID-19 on a cruise ship (Diamond Princess) in late January 2020 and the USS Theodore Roosevelt in March 2020 provide unique opportunities, similar to those provided by the USS Byrd, to gain a more detailed understanding of transmission patterns for SARS-CoV-2. To date, published assessments of COVID-19 outbreaks in these 2 separate settings consist of initial assessments, 1 documenting the occurrence of 700 cases of COVID-19 among nearly 3,700 passengers and crew members in the cruise ship (*38*). The investigation identified that 15 of 20 cases in crew members were in food workers, and 16 of these 20 persons slept in cabins on deck 3. No details were provided for the distribution of COVID-19 cases in passengers, nor of the ventilation system in this cruise ship (*38*). A follow-up assessment was limited to 215 Hong Kong passengers after quarantine and disembarkation; 9 tested positive for SARS-CoV-2 (*39*). No berthing information is available for those passengers. The USS Roosevelt outbreak investigation was a serostudy of a convenience sample of 382 crew members (*40*). Although the sample was not representative of the entire crew, 60% of the participants had antibodies to SARS-CoV-2, indicating prior infection. Notably, 20% of the seropositive group denied having symptoms. Also, as is the case with asymptomatic TB, the degree to which these asymptomatic persons transmitted the infection is not known. Examination of crew member duty rosters and assessment of ventilation patterns in areas inhabited by infected and noninfected persons could provide important information concerning aerosol transmission and the role of spread of the virus by asymptomatic persons. Although the outbreak on the USS Byrd occurred >50 years ago, its assessment is a model for advancing knowledge by thorough investigations, including environmental studies to examine the role of air circulation. With increasing speculation and uncertainty about basic questions such as relative importance of different transmission modes for SARS-CoV-2 (*5*,*7*), the Diamond Princess and USS Roosevelt outbreaks present opportunities, similar to that provided by the USS Byrd, that should not be overlooked.

As noted, although contact identification and evaluation are widely used in high-income, low TB–incidence countries, implementation is limited in low- and middle-income countries. Given the experience with TB, considerable patience, skill, and ingenuity are needed in the implementation of contact tracing for COVID-19. Digital and other automated technologies have been applied to COVID-19 contact tracing in different country settings (*41*,*42*). This new thinking, coupled with innovative tools, will likely hold lessons and examples for improvements in TB prevention and control.

## Avoiding Past Mistakes and Seizing Present Opportunities

In response to COVID-19, countries are having to reassign or recruit and train staff, as well as to establish a robust laboratory diagnostic testing capacity to deliver timely quality-assured results. Early reports from the United States have documented that the COVID-19 response has diverted resources away from essential TB services (*43*). This scenario must be avoided; investments required should be used to improve all public health programs and be sustained over time. Thirty-five years ago, TB provided a dramatic example of the impact of inattention to, and disinvestments in, basic public health programs. During 1985–1992, a reversal of longstanding downward trends occurred as well as and 20% increase in cases (*44*,*45*).

We now have a rare opportunity to seize the moment and use the attention garnered by this novel virus pandemic to ensure that new investments contribute not only to the control of COVID-19, but also to the strengthening of older, yet very relevant public health programs, and to recognize that lessons learned from those programs benefit those at risk for COVID-19. In the United States and in other parts of the world, TB served as the impetus for the establishment of public health programs, and these programs were geared to deal with TB as a public health problem (*46*,*47*). Public health approaches to COVID-19, relying as they do on accelerated responses, digital technologies, and large numbers of trained community-based contact investigators, could establish a new more comprehensive paradigm for the public health programs of the future.
